# *Clostridioides difficile* and Vancomycin-Resistant Enterococci in COVID-19 Patients with Severe Pneumonia

**DOI:** 10.3390/life11111127

**Published:** 2021-10-22

**Authors:** Kateřina Bogdanová, Lenka Doubravská, Iva Vágnerová, Kristýna Hricová, Vendula Pudová, Magdaléna Röderová, Jan Papajk, Radovan Uvízl, Kateřina Langová, Milan Kolář

**Affiliations:** 1 Department of Microbiology, University Hospital Olomouc, I. P. Pavlova 6, 779 00 Olomouc, Czech Republic; katerina.bogdanova@fnol.cz (K.B.); iva.vagnerova@fnol.cz (I.V.); milan.kolar@fnol.cz (M.K.); 2 Department of Anesthesiology, Resuscitation and Intensive Care, University Hospital Olomouc, I. P. Pavlova 6, 779 00 Olomouc, Czech Republic; jan.papajk@fnol.cz (J.P.); radovan.uvizl@fnol.cz (R.U.); 3 Department of Microbiology, Faculty of Medicine and Dentistry, Palacky University Olomouc, Hnevotinska 3, 779 00 Olomouc, Czech Republic; kristyna.hricova@upol.cz (K.H.); vendula.pudova@upol.cz (V.P.); magdalena.roderova@upol.cz (M.R.); 4 Department of Medical Biophysics, Faculty of Medicine and Dentistry, Palacky University Olomouc, Hnevotinska 3, 779 00 Olomouc, Czech Republic; katerina.langova@upol.cz

**Keywords:** COVID-19, *Clostridioides difficile*, oral vancomycin prophylaxis, vancomycin-resistant enterococci, molecular typing of VRE, ICU

## Abstract

Broad-spectrum antibiotics administered to patients with severe COVID-19 pneumonia pose a risk of infection caused by *Clostridioides difficile*. This risk is reduced mainly by strict hygiene measures and early de-escalation of antibiotic therapy. Recently, oral vancomycin prophylaxis (OVP) has also been discussed. This retrospective study aimed to assess the prevalence of *C. difficile* in critical COVID-19 patients staying in an intensive care unit of a tertiary hospital department of anesthesiology, resuscitation, and intensive care from November 2020 to May 2021 and the rates of vancomycin-resistant enterococci (VRE) after the introduction of OVP and to compare the data with those from controls in the pre-pandemic period (November 2018 to May 2019). During the COVID-19 pandemic, there was a significant increase in toxigenic *C. difficile* rates to 12.4% of patients, as compared with 1.6% in controls. The peak rates were noted in February 2021 (25% of patients), immediately followed by initiation of OVP, changes to hygiene precautions, and more rapid de-escalation of antibiotic therapy. Subsequently, toxigenic *C. difficile* detection rates started to fall. There was a nonsignificant increase in VRE detected in non-gastrointestinal tract samples to 8.9% in the COVID-19 group, as compared to 5.3% in the control group. Molecular analysis confirmed mainly clonal spread of VRE.

## 1. Introduction

For coronavirus disease 2019 (COVID-19) patients with critical pneumonia requiring high-flow oxygen therapy (HFOT), mechanical ventilation, or even extracorporeal membrane oxygenation (ECMO), broad-spectrum antibiotic therapy is recommended by the World Health Organization (WHO) [1]. However, the role of antibiotic therapy in these most severe patients remains unclear. Studies published so far suggest that the incidence of secondary bacterial infections is lower than originally expected based on previous respiratory disease epidemics (severe acute respiratory syndrome, Middle East respiratory syndrome, H1N1 influenza) [2,3]. Questions persist as to whether antibacterial therapy should be immediately initiated in COVID-19 inpatients requiring invasive oxygen therapy, mechanical ventilation, or ECMO.

Administration of broad-spectrum antibiotics, often necessary in critical COVID-19 patients suspected of bacterial superinfection, poses a high risk for *Clostridioides difficile* infection (CDI), with some antibiotic classes being more strongly associated with CDI [4]. Several reports have already been published concerning CDI occurrence during the COVID-19 pandemic with contradictory findings: a decrease or stable number of cases [5,6,7,8,9] as opposed to a minor [10] or significant [11] increase in the incidence of CDI. Some authors also admitted or warned against the possibility of underdiagnosing CDI during the COVID-19 pandemic, as the gastrointestinal symptoms and diarrhea typical for CDI could have been attributed to COVID-19 [8,11].

CDI is growing in incidence in Europe and USA and contributes to morbidity and mortality [12,13] despite the efforts to reduce this healthcare-associated infection, mostly by effective antimicrobial stewardship and strategies that are necessary to prevent the spread of bacterial spores. However, the main CDI risk factor, administration of antibiotics, remains necessary for patient treatment. Another possible strategy for preventing CDI, antibiotic prophylaxis such as the application of oral vancomycin, has been evaluated in several studies with mixed results [14]. The use of oral vancomycin prophylaxis may also be seen as controversial, as it can potentially lead to the selection of vancomycin-resistant enterococci (VRE) and gut dysmicrobia that by itself increases the risk of *C. difficile* and other multidrug-resistant (MDR) bacteria colonization even weeks after vancomycin administration [15].

The present study aimed to analyze detection of *C. difficile* and VRE in hospital patients with severe COVID-19 requiring HFOT or mechanical ventilation.

## 2. Materials and Methods

### 2.1. Study Design, Population and Setting

The study was designed as observational and retrospective and performed in the intensive care unit (ICU) of the Department of Anesthesiology, Resuscitation and Intensive Care (KARIM), University Hospital Olomouc and Faculty of Medicine and Dentistry, Palacky University Olomouc. The study group comprised patients hospitalized over a 7-month period during the COVID-19 pandemic. Individuals staying in KARIM ICU over the same seven months before the pandemic were used as controls. Ethics committee approval was not needed as all patients underwent standard diagnostic and therapeutic procedures.

The University Hospital Olomouc is a 1200-bed tertiary medical center with a 10-bed general KARIM ICU providing the highest level of intensive care. The unit consists of one suite with four private beds and six in an open space. During the COVID-19 pandemic, the capacity of the department was extended to as many as 4 to 5 adjacent suites with up to 35 beds (350% of the normal capacity), mostly (approximately 70%) in the open-space arrangement, connected by doors or hallways with shared staff changing and equipment rooms.

### 2.2. Inclusion Criteria

The study group comprised patients admitted to KARIM ICU between 1 November 2020 and 31 May 2021 for critical COVID-19 with acute respiratory failure and need for HFOT or mechanical ventilation, receiving initial antibiotic treatment based on combination cefotaxime or amoxicillin/clavulanic acid with clarithromycin. The control group included patients with acute respiratory failure and need for HFOT or mechanical ventilation admitted to KARIM ICU between 1 November 2018 and 31 May 2019, that is, before the COVID-19 pandemic. Patient data were obtained from electronic medical records.

### 2.3. Detection of C. difficile (Positive Cases)

Stool samples, or deep rectal swabs in the case of patients with gastrointestinal tract (GIT) paralysis, were tested for *C. difficile* antigen (glutamate dehydrogenase) and toxins A/B (Rapid-VIDITEST, Vidia, Vestec, Czech Republic). Positive cases were patients with positive *C. difficile* antigen and/or toxin. Due to the increasing frequency of positive cases, most ICU patients were tested for the presence of *C. difficile* after 1 March 2021.

### 2.4. Isolation of VRE

Clinical samples (tracheal aspirate, urine, blood) were collected from all patients staying in KARIM ICU to isolate potential pathogens, including VRE. All samples were inoculated onto culture agar plates (TRIOS, Praha, Czech Republic) and processed by standard procedures. Starting from April 2021, patients tested for *C. difficile* were parallelly tested for VRE colonization. Stool and rectal swab samples were collected and inoculated onto selective media for the isolation of VRE (Brilliance VRE Agar, Oxoid, Brno, Czech Republic). Species identification of isolates was carried out by MALDI-TOF MS (Biotyper Microflex, Bruker Daltonics, Billerica, MA, USA). From each patient, only one isolate identified as the first one from one type of clinical sample was included. Resistance to vancomycin was detected by the microdilution method according to the European Committee on Antimicrobial Susceptibility Testing (EUCAST). *Enterococcus faecalis* ATCC 29212 reference strain was used for quality control. Resistance to vancomycin was confirmed by detecting the relevant resistance genes as described by Hricova et al. [16]. Isolated strains were stored in cryotubes at −80 °C (Cryobank B, ITEST, Hradec Kralove, Czech Republic).

### 2.5. Molecular Typing of VRE

Molecular typing of all isolated VRE was performed using pulsed-field gel electrophoresis (PFGE) as described by Hricová et al. [16]. The coefficient of similarity (CS) was calculated using the Dice algorithm. Individual clusters were analyzed with the unweighted pair group method with arithmetic mean algorithm and the results were interpreted using criteria defined by Tenover et al. [17]. Optimization and band matching tolerance was set at 1.5%. Restriction profiles reaching 95% similarity were considered identical.

### 2.6. Vancomycin Consumption Assessment

For the two study periods, the data on vancomycin use were extracted from hospital electronic records. Data are expressed as defined daily doses (DDD_ATB_) according to the criteria of the WHO Collaboration Centre for Drug Statistics Methodology—the 2020 ATC/DDD system [18] (WHO).

### 2.7. Statistical Analysis

Qualitative data were compared using the χ^2^ test. The Mann–Whitney U-test was used to compare ages in the groups. Statistical analyses were performed with IBM SPSS Statistics for Windows, version 23.0 (Armonk, NY, USA: IBM Corp.).

## 3. Results

### 3.1. Patient Data Analysis—The Study and Control Groups

During the COVID-19 pandemic, the number of patients staying in KARIM ICU was nearly double (196.8%) the average. There were no statistically significant differences in age, gender, primary and secondary admissions, or mortality between the COVID-19 group (2020–2021) and pre-pandemic controls (2018–2019). The characteristics of both groups including statistical data are shown in Table 1.

### 3.2. C. difficile Detection

**Figure 1:**
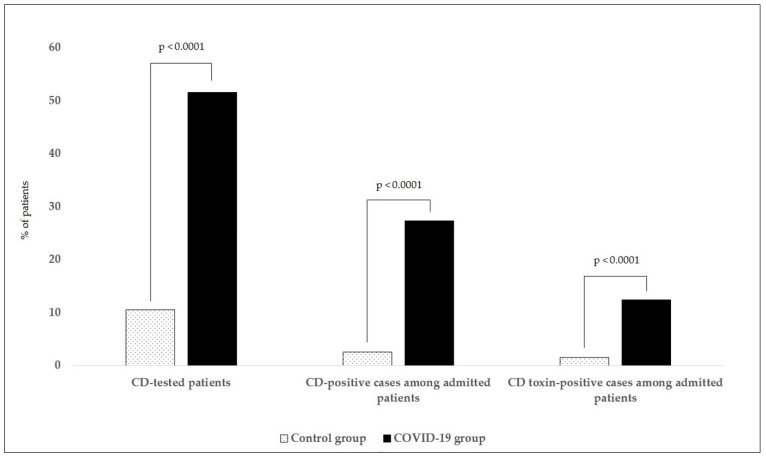
*C. difficile* statistical analysis. We compared the numbers of performed *C. difficile* detection tests and the numbers *C. difficile* positive cases (antigen-positive) and *C. difficile*-toxin positive cases among hospitalized patients. We detected a significant increase (*p* < 0.0005) in COVID-19 compared to the control group in all three aspects (number of performed tests, number of CD positive cases, and number of positive CD-toxins). CD: *Clostridioides difficile*.

The results of *C. difficile* detection and statistical analysis are shown in Table 2 and Figure 1, respectively. Over the pre-pandemic period (1 November 2018 to 31 May 2019), CDI was clinically suspected and tested in 20 out of 189 hospitalized patients (10.6%). In five cases (2.6%), the presence of *C. difficile* was confirmed and toxin production was found in three patients (1.6%). There was a significant increase in the number of *C. difficile* detection tests performed (*p* < 0.0001) during the pandemic (1 November 2020 to 31 May 2021)—192 out of 372 patients (51.6%) were tested for the presence of *C. difficile*, with 102 cases (27.4%) being positive. Toxin production was detected in 46 patients (12.4%). Both positive cases of *C. difficile* detection and toxin detection were significantly higher compared to the control group (*p* < 0.0001). Taking into consideration all risk factors and the possibility of higher exogenous spread of bacterial spores, the frequency of *C. difficile* examination increased considerably. The proportion of patients tested rose to 62.5% in February and more than 80% in the following months (March to May 2021). A time analysis (Table 2) revealed that the detection rates of toxigenic *C. difficile* started to elevate in January (14.4% of patients) and peaked in February 2021 (25% of patients).

As a result, apart from therapeutic doses of oral vancomycin 125 mg four times daily (also occasionally combined with metronidazole 500 mg three times daily intravenously) given to patients positive for *C. difficile* toxin or suspected of CDI, prophylactic doses of oral vancomycin 125 mg once daily were administered to the other hospitalized patients. Moreover, organizational precautions were implemented in the department and initial broad-spectrum antibiotics were more rapidly de-escalated as suggested by newly developed guidelines on antibiotic therapy of COVID-19 patients. Subsequently, the *C. difficile* rates dropped to 30.8% in April and 33.3% in May 2021. Similarly, the percentages of toxigenic strains decreased to 13.5% and 9.5%, respectively.

### 3.3. VRE Detection

Table 3 documents the development over time of the frequency of VRE detected in various clinical samples obtained from critical COVID-19 patients compared to the pre-pandemic period, without VRE isolated from the GIT. Figure 3 shows statistical analysis. If VRE were detected in various clinical samples collected from a single patient, that patient was counted as one only.

**Figure 2:**
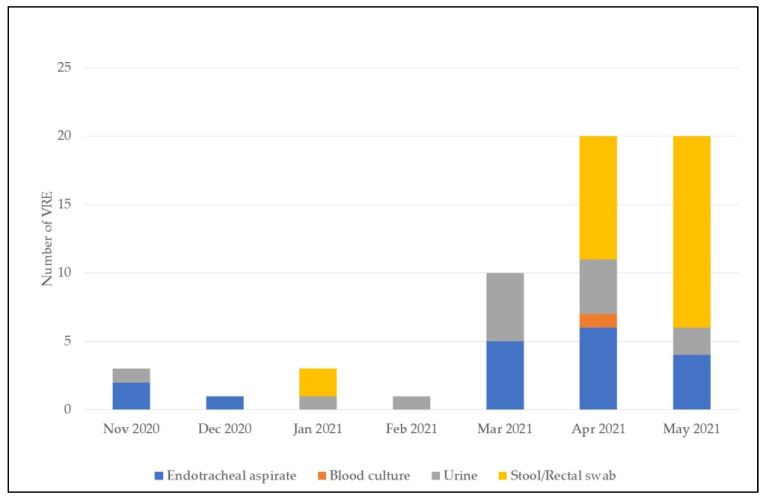
VRE cases between 1 November 2020 and 31 May 2021. For April and May, when the VRE screening was performed, the total number of detected VRE is shown, including VRE detected in both GIT and non-GIT samples collected from the same patient.

In the pre-pandemic period, a total of 10 VRE were isolated from endotracheal aspirate, urine, and positive blood cultures (Table 3). During the COVID-19 pandemic, 33 VRE were detected in non-GIT samples and 25 in samples obtained from the GIT (Table 3 and Figure 2). From November 2020 to March 2021, the positive VRE detection was mainly from endotracheal aspirate and urine. In March 2021, the rate of VRE detection was considerably higher than in the previous months. Therefore, a decision was made to start active VRE screening using patient stool and rectal swab samples during April and May 2021. The increase in VRE detection in April and May was mainly attributable to positive samples from the GIT. However, even after excluding the GIT-VRE positivity, the proportion of VRE-positive patients was 21.2% and 28.6% in April and May, respectively. Only 7 out of 21 patients admitted in May were not infected or colonized with VRE.

**Figure 3:**
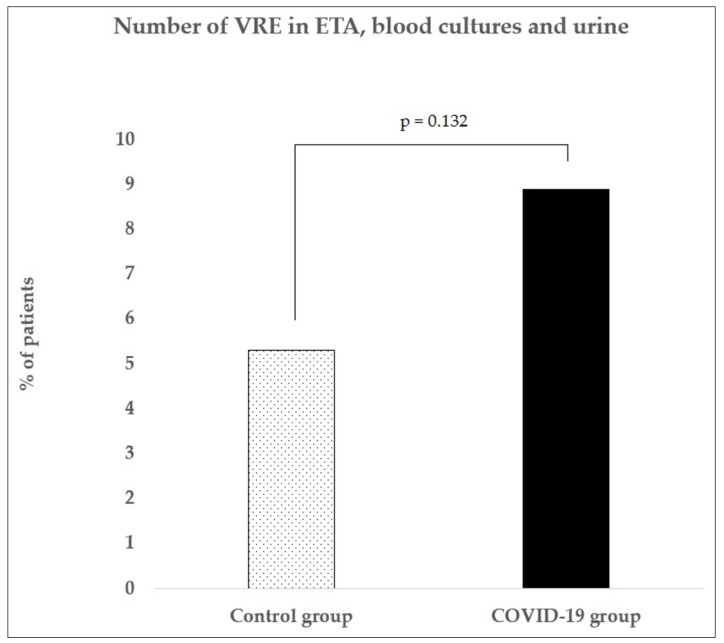
Vancomycin-resistant enterococci (VRE) statistical analysis. We performed the statistical analysis of VRE detected in non-GIT: endotracheal aspirate, blood cultures, and urine. According to our findings, there was no statistically significant increase in the COVID-19 group compared to the control group (*p* = 0.132). VRE: vancomycin-resistant enterococci; ETA: endotracheal aspirate.

Because VRE screening was not performed in the control group, only non-GIT VRE-positive cases were compared to the COVID-19 group and statistically evaluated (Figure 3). There was a slight, nonsignificant increase in VRE incidence between the control and COVD-19 groups (*p* = 0.132).

### 3.4. Molecular Typing of VRE

**Figure 4:**
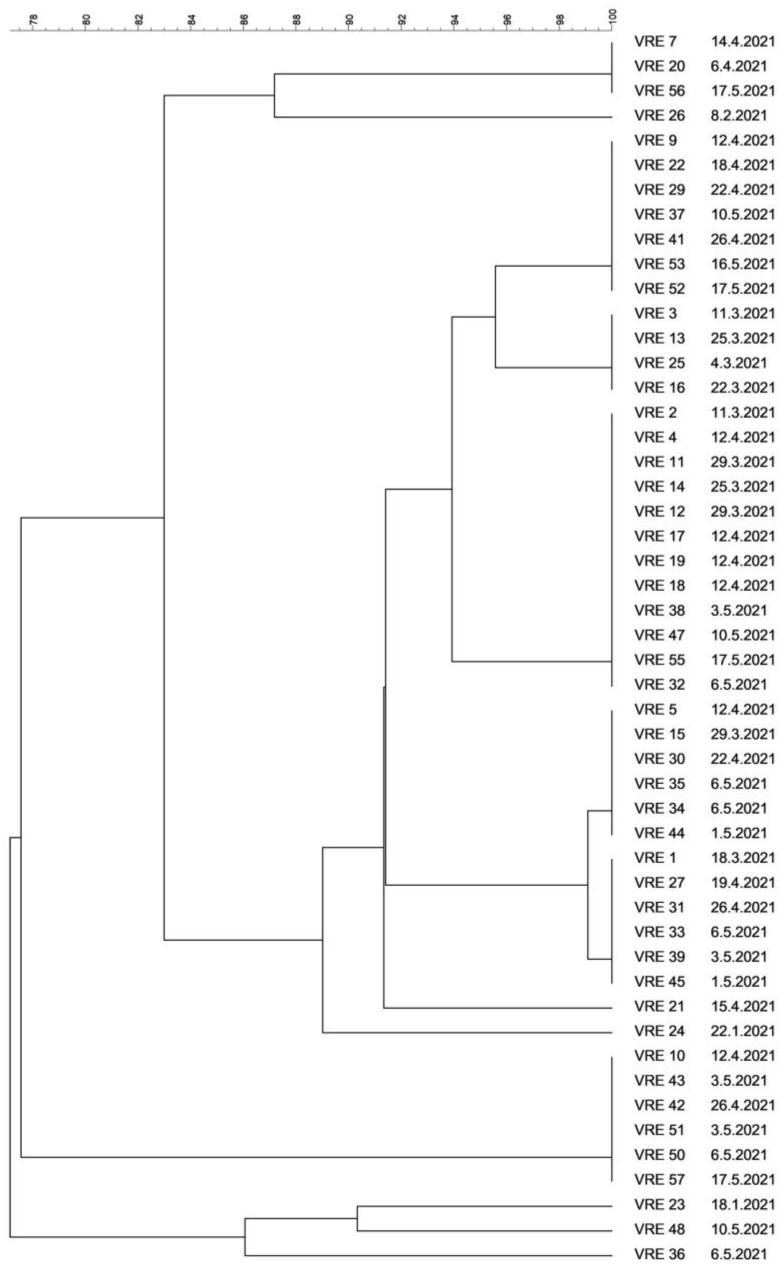
Dendrogram of 50 vancomycin-resistant *Enterococcus faecium* isolates.

**Figure 5:**
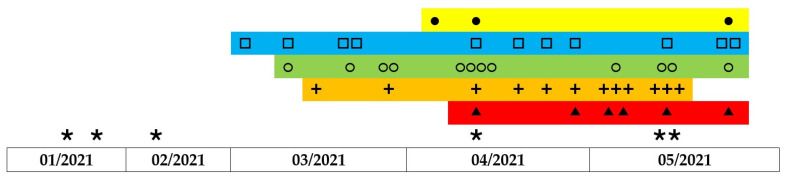
Timeline of the prevalence of *VRE* clones throughout the study duration. The grey rectangles represent 5 clonal groups of vancomycin-resistant *Enterococcus faecium* based on their similarity. Individual isolates are represented by the symbols ●□○+▲ within clusters. Six strains with unique profiles are marked with ∗.

VRE for molecular typing were collected from January to May 2021. In all VRE-positive patients hospitalized during the COVID-19 pandemic, a strain of *Enterococcus faecium* was isolated; the *vanA* gene was found in all isolates while the *vanB* gene was not detected. Similarity or identity was assessed in 50 isolates of VRE; three isolates could not be cultured again. Comparison of whole-genome DNA restriction profiles revealed identical or very similar restriction profiles, suggestive of clonal spread. In five patients, identical VRE isolated were obtained from various samples; therefore, only one isolate from each patient was included. In one case, a VRE strain isolated from a rectal swab was different from that detected in sputum so both were included. Only six (12%) out of 50 isolates had unique restriction profiles; the remaining 44 were divided into five groups based on comparison of their macrorestriction profiles. PFGE revealed various sizes of the clonal groups, with 12, 12, 11, 6, and 3 isolates. The results of strain comparison by PFGE are shown in Figure 4. Twelve, six, and three identical isolates showed a CS of 100%, whereas 12 and 11 isolates showed a CS exceeding 95% and were thus considered identical as well. The timeline of the prevalence of VRE clones throughout the study duration is shown in Figure 5.

### 3.5. Vancomycin Consumption

Vancomycin consumption was shown to increase from 103 DDD_ATB_ in the pre-pandemic period to 169 DDD_ATB_ during the COVID-19 pandemic. However, the percentage of vancomycin among all antibacterials administered remained roughly the same (2.9% and 2.3%, respectively).

## 4. Discussion

Intensive care patients have several risk factors for the development of CDI, namely antibiotic therapy, intestinal dysmotility, immobility, older age, stress ulcer prophylaxis with proton pump inhibitors resulting in low stomach acid, and comorbidities. Their level of mucosal immunity may also play a role [19]. According to Zuo et al. [20] SARS-CoV-2 infection results in gut microbiome disruption with an increase in opportunistic pathogens and depletion of beneficial commensals persisting even after clearance of virus.

The present study aimed to analyze the presence of *C. difficile* and VRE in patients with severe COVID-19. Compared with pre-pandemic controls, there was a significant increase in detection of *C. difficile* that started in January 2021 and peaked in February (40% of patients) and March 2021 (47.6% of patients). Toxigenic strains of *C. difficile* were detected in 25% and 13.3% of patients in February and March 2021, respectively. In the following months, *C. difficile* rates slowly decreased; at the end of the study in May 2021, the toxin was confirmed in 9.5% of patients. With the increasing prevalence of *C. difficile*, numerous measures were implemented in the department, namely faster de-escalation of antibiotic therapy, hygiene measures, and primary prophylaxis with oral vancomycin at a dose of 125 mg once daily.

Infections caused by *C. difficile* during the COVID-19 pandemic have been investigated by many authors [5,6,7,8,9,10,11,21]. Some, for example, Allegretti et al. [9] and Hazel et al. [21], reported lower rates of CDI, attributing them to the implementation of strict hygiene precautions as well as to the isolation of patients with CDI, which was impossible in our department during the COVID-19 pandemic. A study by Lewandowski et al. [11] is one of few to describe a significant increase in CDI, stressing that the disease may be underdiagnosed. In the aforementioned studies, CDI tests were performed due to repeated diarrhea. However, gastrointestinal symptoms are also present in COVID-19 and CDI may not always have been suspected.

While most symptomatic COVID-19 patients present with fever, cough, shortness of breath, and/or loss of the sensation of taste and smell, according to Up-To-Date [22], up to one-third of patients present with gastrointestinal complaints, including critical patients with acute respiratory distress syndrome (ARDS) requiring intensive care. Published systematic reviews suggest that diarrhea is seen in 11.5% to 12.5% of COVID-19 patients [23,24]. Another systematic review found high variability of diarrhea prevalence in COVID-19 patients, ranging from 2% to 50% [25].

Although the most prominent and frequent clinical sign of CDI is diarrhea, the symptoms may range from mild gastrointestinal discomfort to severe pseudomembranous colitis and to toxic megacolon with paralytic ileus [26]. Gastrointestinal motility disorders which include gastroparesis, ileus, and acute colonic pseudo-obstruction are common in critically ill ICU patients [27], another possible reason for CDI underdiagnosis among these patients. The issue of underdiagnosing *C. difficile* was highlighted by Davies et al. [28]. As in the present study, GIT paralysis was found in the majority of patients, the diagnosis of CDI could not rely on the presence of diarrhea as the main symptom of the disease; on the contrary, paralysis had to be considered as a potential symptom and these patients had to be examined. This was also one of the reasons for an increase in the number of tests performed (over 80% in the last three months of the study). From patients with GIT paralysis, samples were collected by deep rectal swabs. We must admit that more frequent detection of *C. difficile* resulted from the greater numbers of tests.

A known risk factor for the development of CDI is the use of broad-spectrum antibiotics. In COVID-19 patients, it is recommended to administer antibiotics when bacterial coinfection is suspected and then de-escalate antibiotic therapy as early as possible based on microbiology tests [29,30]. This approach stems from concerns about delayed antibiotic therapy with potentially serious consequences including the development of sepsis and death [31,32,33].

Opinions on antibiotic therapy in patients with critical COVID-19, that is, with ARDS or sepsis or septic shock, have gradually developed. However, according to recent data, only 3% to 7% of patients with COVID-19 staying in general wards have community-acquired bacterial coinfections [34,35,36,37,38,39]. In ICUs, the proportion is reported to be higher, ranging from 14% to 28% [40,41]. It seems that while community-acquired coinfections are not so common in critical COVID-19 patients, the risk for bacterial nosocomial superinfection is increased due to many factors (prolonged hospital stay, mechanical ventilation, inserted invasive devices, but also immunosuppression with frequent lymphopenia).

Despite these results, many studies have documented the administration of antibiotics to more than 50% of hospitalized patients. The lowest proportion (43%) was reported by Aggarwal et al. [42]. Most studies, however, state that antibiotics are administered to 60% to 90% of patients [43,44,45,46,47,48] or even all patients [49,50,51]. Two meta-analyses [35,36] found that on average, 72% of patients received antibiotics. The duration of antibiotic therapy is rarely specified in studies [49,52,53]. The above studies mention the use of cephalosporins, fluoroquinolones including moxifloxacin and macrolides, mostly azithromycin. Other antibiotics were less frequently administered.

Recommendations from both international and national societies are rather general. For example, the Surviving Sepsis Campaign statement entitled “Guidelines on the management of critically ill adults with Coronavirus Disease 2019 (COVID-19)” [54] recommends that in mechanically ventilated patients with COVID-19 and respiratory failure, empiric antimicrobials/antibacterial agents should only be administered if bacterial coinfection is suspected. However, no specific antibiotic regimen is stated there. According to German National Guidelines, empiric broad-spectrum antibiotic therapy should be started as soon as possible in patients suspected to have bacterial superinfection [55]. Some national societies suggest that the approach should be identical to that in common community-acquired pneumonia [29,30].

In the present study, the group of critical COVID-19 patients comprised 50% of primary admissions (hospital stay of less than 48 h). Here, a small proportion of bacterial coinfections could surely be expected. In these clinically very complicated cases, however, intensive care clinicians have a very hard time initially recognizing whether bacterial superinfection is present, without knowing microbiology test results. In that situation, the recommendations are consistent. Antibiotic therapy is initiated when bacterial pneumonia is clinically suspected and discontinued if microbiology tests yield negative results. Therefore, nearly all our patients were initially treated with antibiotics. This could have been one of the factors contributing to the increased detection of *C. difficile.* In this group of patients, the incidence of common bacterial pathogens was low but atypical pathogens were more frequent. The other half of the patients, those transferred to our department after staying in another department or hospital for more than 48 h, were already at risk for healthcare-associated pneumonia and thus received appropriate care. Changes in the spectrum of pathogens causing healthcare-associated pneumonia and their resistance to antibiotics in critical COVID-19 patients are addressed in another original article.

Recommendations for initial therapy of COVID-19 patients including critically ill individuals in ICUs were gradually updated as more evidence was available. This resulted in, among others, shorter duration of antibiotic therapy and earlier de-escalation which may have contributed to the later decrease in *C. difficile* rates.

Another important factor contributing to increased detection of *C. difficile* was the overall situation in the KARIM ICU and hospital alike. During the COVID-19 pandemic, bed transformation was performed at all levels of care. The capacity of ICU beds was increased, partly by transforming other wards and units and partly by creating new ones. Day-to-day adjustments to the construction and equipment of the units could not be made so it was impossible to provide enough private beds necessary for the isolation of patients with *C. difficile*. The staff of the extended ICU consisted of professionals working in ICUs, an emergency department, or general wards, as well as medical students and temporary external workers. During the pandemic, the number of KARIM ICU beds rose 3.5-fold compared to the usual capacity. At that time, the situation was unique in that all patients were admitted for coronavirus positivity and the overwhelming majority (97.5%) were those with critical COVID-19 manifestations as defined by the WHO [1], that is, severe course of the disease with clinical manifestations of ARDS, sepsis, or septic shock.

The higher numbers of both patients and staff members meant increased movement of people throughout the department. The staff comprised workers from other departments not completely acquainted with their new workplace. Working in protective equipment was considerably more difficult, potentially leading to unnoticed contamination of coveralls or other aids. Under such circumstances, sterility is difficult to maintain during all invasive procedures, infusion preparation, and other activities. Moreover, one of the key therapeutic procedures provided to patients with critical COVID-19 pneumonia is regular prone and supine positioning. This maneuver is performed twice daily and requires approximately six health workers directly touching the patient and devices.

Once the rise of *C. difficile* positive cases was noticed, an inspection was performed by workers from the hospital hygiene department and the regional public health authority, followed by implementation of the following measures. Efforts were made to reserve one unit for this group of patients due to a lack of cubicles. All premises were treated with sporicidal agents. The staff were re-trained in hygiene issues, focusing on the prevention of *C. difficile* transmission. The workers wore an additional piece of protective clothing, a disposable plastic apron. The frequency of surface cleaning was increased, and disinfectants were replaced with agents active against *C. difficile* spores. After contact with each patient, the staff wiped their coveralls with soapy water and dried them thoroughly, as stated in general recommendations [56].

Given the significant increase in toxigenic strains of *C. difficile* in February (25%), primary oral vancomycin prophylaxis (OVP) was implemented in the department, with a dose of 125 mg once daily being administered to all patients not receiving oral vancomycin at a therapeutic dose of 125 mg four times daily or other anti-*C. difficile* antibiotics. The administration of OVP has been investigated in numerous studies. It was reported as effective by Johnson et al. [57] and Ganetsky et al. [58]. In those two retrospective studies, no CDI was detected in patients receiving OVP. Similarly, a meta-analysis by Babar et al. [59] documented a reduction in CDI in high-risk patients following OVP administration. In their meta-analysis, Tariq et al. [14] suggested a positive effect of vancomycin to prevent CDI recurrence (secondary prophylaxis). However, there was no benefit for primary prevention. Another issue is the dosage of OVP; the most common dose was 125 mg twice daily, but doses of 125–250 mg four times daily or 125 mg once a day were also administered [57,58,60,61,62,63,64,65]. Our decision was to administer 125 mg once daily, assuming that the dose would ensure sufficient vancomycin concentration in stools, with minimum damage to the intestinal flora and subsequent reduction of the resistance of the GIT to colonization with multidrug-resistant bacteria including *C. difficile* [66,67,68,69,70,71].

We were aware of the risk of selection pressure affecting the prevalence of VRE. Prior to the initiation of OVP, we detected six VRE strains in endotracheal aspirate and urine samples, a rate similar to that in the pre-pandemic period (5 VRE strains). In March 2021, when OVP administration was started, slightly more VRE (10 strains) were detected in extra-intestinal samples, which prompted active screening for VRE in samples collected from the GIT. In April and May, 17 and 23 VRE were detected in non-GIT and GIT samples, respectively. When compared to the control group, the VRE numbers in non-GIT materials were slightly, but not significantly, elevated in the COVID-19 group. Similarly, three of the above studies on OVP monitored newly detected VRE but none reported their significant increase [57,58,64].

It may be stated that the present study found an increase in VanA phenotype VRE of *Enterococcus faecium*, particularly in GIT samples. When calculating isolates obtained from the GIT only, as many as 66.7% of patients were identified as VRE carriers in May 2021. However, this was mainly due to the clonal spread of VRE. PFGE revealed several groups of isolates with identical profiles or very high coefficients of similarity (>95%), suggesting very likely clonal spread and transmission of VRE among patients. As the difference in restriction profiles between the tree largest clusters was very small (CS > 91%), isolates in these groups may be assumed to be closely related, similar to a study by Tenover et al. [17]. Genetically related VRE isolates in COVID-19 patients in the ICU were also reported by Kampmeier et al. [72]. Their study describes the genetic relationship of VRE isolates collected from patients and several strains isolated from the environment, stressing possible surface contamination. The important role played by the environment in VRE transmission in hospitals was noted by Correa-Martinez [73] and others.

There are several limitations to our work. The character of the study is retrospective. There was considerable heterogeneity between the control and COVID-19 groups of patients. As already mentioned, the situation in KARIM ICU was constantly evolving during the COVID-19 pandemic, including changes in antibiotic therapy, hygiene strategies, and frequency of *C. difficile* and VRE detection tests performed, so the conditions of the study were highly variable. We were also unable to perform any kind of VRE follow-up of discharged patients. This could have led to an underestimation of VRE colonization. Moreover, only the number of VRE detected in non-GIT samples in the COVID-19 group could be weighed against the control group because of the absence of screening performed at that time. In the case of *C. difficile*, we did not isolate the bacterial strains for molecular typing to further investigate clonal similarities, which could have possibly allowed us to assess whether the source of *C. difficile* infection/colonization was mainly exogenous or endogenous.

## 5. Conclusions

During the COVID-19 pandemic, there was a significant increase in *C. difficile* in KARIM ICU. This was likely due to not only the initial administration of broad-spectrum antibiotic agents but also, unfortunately, the limited possibility to adhere to strict hygiene measures in that situation. The highest rates of toxigenic *C. difficile*, noticed in February 2021, started to decline in March. This may be attributed to several factors: earlier diagnosis of *C. difficile* (screening), adjustments and adherence to hygiene and epidemiological measures, earlier de-escalation of broad-spectrum antibiotic therapy, and prophylactic administration of vancomycin. The exact contribution of individual measures to *C. difficile* reduction cannot be defined accurately. During the administration of oral vancomycin prophylaxis, higher VRE rates were noted, mainly due to the clonal spread of these strains. It may be assumed that vancomycin prophylaxis caused the selection of VRE already present in the GIT and their subsequent clonal spread. However, it cannot be definitely concluded that vancomycin prophylaxis alone results in an increased prevalence of vancomycin-resistant enterococci.

## Figures and Tables

**Table 1:** Characteristics and statistical analysis of the pre-pandemic and COVID-19 groups.

Variable	Control Group 2018–2019 (n = 189)	COVID-19 Group 2020–2021 (n = 372)	*p*-Value
Gender—males (%)	136 (72)	241 (64.2)	0.087
Age (median, range, IQR)	65, 18–97, 20	67, 22–90, 16	0.753
Admission up to 48 h after admission to hospital (%)	82 (43.4)	186 (50)	0.138
Transfer from another hospital or department	107 (56.6)	186 (50)	0.138
No. of patients on mechanical ventilation only	180 (95.2)	78 (21.0)	<0.0001
No. of patients on HFOT only	3 (1.6)	112 (30.1)	<0.0001
No. of patient on both mechanical ventilation and HFOT	6 (3.2)	182 (48.9)	<0.0001
No. of patients on ECMO	0 (0.0)	24 (6.5)	0.004
ICU mortality	74 (39.2)	158 (42.5)	0.450

HFOT: high-flow oxygen therapy; ECMO: extracorporeal membrane oxygenation; IQR: interquartile range.

**Table 2:** *C. difficile* antigen and toxin detection and number of tests performed before and during the COVID-19 pandemic.

Month	Control Group 2018–2019				COVID-19 Group 2020–2021			
	No. of Admitted Patients	No. (%) of CD-Tested Patients	No. (%) of CD-Positive Cases among Admitted Patients	No. (%) of CD Toxin-Positive Cases among Admitted Patients	No. of Admitted Patients	No. (%) of CD-Tested Patients	No. (%) of CD-Positive Cases among Admitted Patients	No. (%) of CD Toxin-Positive Cases among Admitted Patients
November	23	2 (8.7)	0	0	55	8 (14.5)	3 (5.5)	2 (3.6)
December	28	2 (7.14)	0	0	56	7 (12.5)	5 (8.9)	3 (5.4)
January	30	4 (13.3)	1 (3.3)	1 (3.3)	64	20 (31.2)	15 (23.4)	9 (14.1)
February	35	5 (14.3)	1 (2.9)	1 (2.9)	40	25 (62.5)	16 (40)	10 (25)
March	28	3 (10.7)	1 (3.6)	0	84	71 (84.5)	40 (47.6)	13 (15.5)
April	22	2 (9.1)	2 (9.1)	1 (4.5)	52	44 (84.6)	16 (30.8)	7 (13.5)
May	23	2 (8.7)	0	0	21	17 (81)	7 (33.3)	2 (9.5)
Total	189	20 (10.6)	5 (2.6)	3 (1.6)	372	192 (51.6)	102 (27.4)	46 (12.4)

CD: Clostridioides difficile.

**Table 3:** VRE detected in various clinical samples, excluding GIT samples, collected from the pre-pandemic and COVID-19 groups. The absolute numbers do not include repeatedly detected VRE (VRE detected in multiple clinical samples from a single patient were counted as one only).

Month	Control Group 2018–2019					COVID-19 Group 2020–2021				
	No. of Admitted Patients	No. of VRE in ETA	No. of VRE in Blood Cultures	No. of VRE in Urine	Absolute no. (%) of VRE-Positive Patients	No. of Admitted Patients	No. of VRE in ETA	No. of VRE in Blood Cultures	No. of VRE in Urine	Absolute no. (%) of VRE-Positive Patients
November	23	2	0	1	3 (13.1)	55	2	0	1	3 (5.5)
December	28	0	0	0	0	56	1	0	0	1 (1.8)
January	30	1	1	0	2 (6.7)	64	0	0	1	1 (1.6)
February	35	0	0	0	0	40	0	0	1	1 (2.5)
March	28	3	1	0	4 (14.3)	84	5	0	5	10 (11.9)
April	22	0	0	0	0	52	6	1	4	11 (21.2)
May	23	0	1	0	1 (4.3)	21	4	0	2	6 (28.6)
Total	189	6	2	1	10 (5.3)	372	18	1	14	33 (8.9)

VRE: vancomycin-resistant enterococci; ETA: endotracheal aspirate.

## Data Availability

All data presented in this study are included in this article.
